# NH‐sulfoximine: A novel pharmacological inhibitor of the mitochondrial F_1_F_o_‐ATPase, which suppresses viability of cancerous cells

**DOI:** 10.1111/bph.15279

**Published:** 2020-12-14

**Authors:** Daniela Strobbe, Rosalba Pecorari, Oriana Conte, Antonella Minutolo, Christine M. M. Hendriks, Stefan Wiezorek, Danilo Faccenda, Rosella Abeti, Carla Montesano, Carsten Bolm, Michelangelo Campanella

**Affiliations:** ^1^ Department of Comparative Biomedical Sciences The Royal Veterinary College, University of London London UK; ^2^ Department of Cell and Developmental Biology, Consortium for Mitochondrial Research (CfMR) University College London London UK; ^3^ Department of Biology University of Rome “Tor Vergata” Rome Italy; ^4^ Institute of Organic Chemistry RWTH Aachen University Aachen Germany; ^5^ Ataxia Centre, Department of Clinical and Movement Neurosciences UCL Queen Square Institute of Neurology Queen Square London, London WC1N 3BG UK

**Keywords:** Autophagy, cell death, F_1_F_o_‐ATPsynthase, IF_1_, mitochondria

## Abstract

**Background and Purpose:**

The mitochondrial F_1_F_o_‐ATPsynthase is pivotal for cellular homeostasis. When respiration is perturbed, its mode of action everts becoming an F_1_F_o_‐ATPase and therefore consuming rather producing ATP. Such a reversion is an obvious target for pharmacological intervention to counteract pathologies. Despite this, tools to selectively inhibit the phases of ATP hydrolysis without affecting the production of ATP remain scarce. Here, we report on a newly synthesised chemical, the NH‐sulfoximine (NHS), which achieves such a selectivity.

**Experimental Approach:**

The chemical structure of the F_1_F_o_‐ATPase inhibitor BTB‐06584 was used as a template to synthesise NHS. We assessed its pharmacology in human neuroblastoma SH‐SY5Y cells in which we profiled ATP levels, redox signalling, autophagy pathways and cellular viability. NHS was given alone or in combination with either the glucose analogue 2‐deoxyglucose (2‐DG) or the chemotherapeutic agent etoposide.

**Key Results:**

NHS selectively blocks the consumption of ATP by mitochondria leading a subtle cytotoxicity associated via the concomitant engagement of autophagy which impairs cell viability. NHS achieves such a function independently of the F_1_F_o_‐ATPase inhibitory factor 1 (IF1).

**Conclusion and Implications:**

The novel sulfoximine analogue of BTB‐06584, NHS, acts as a selective pharmacological inhibitor of the mitochondrial F_1_F_o_‐ATPase. NHS, by blocking the hydrolysis of ATP perturbs the bioenergetic homoeostasis of cancer cells, leading to a non‐apoptotic type of cell death.

**Abbreviations**
2‐DG: 2‐deoxyglucose
BTB: BTB‐06584
IAA: iodoacetic acid
IF_1_
: ATPase inhibitory factor 1
MgG: magnesium green
MM: mitochondrial matrix
NHS: NH‐sulfoximine
OMM: outer mitochondrial membrane
TMRM: tetramethylrhodamine methyl ester

**What is already known**

- In cancer cells the mitochondrial F_1_F_o_‐ATPsynthase runs as an F_1_F_o_‐ATPase thereby consuming ATP.

**What this study adds**

- The pharmacological agent, NH‐sulfoximine (NHS) counteracts the F_1_F_o_‐ATPase selectively targeting mitochondria which consume ATP.

**What is the clinical significance**

- Through this mechanism, NHS increases the effects of chemotherapeutic agents, without added toxicity.

## 1 INTRODUCTION

In eukaryotic cells, ATP is preferentially produced by mitochondria through oxidative phosphorylation (OXPHOS), which is dependent on the activity of the multimeric enzyme F_1_F_o_‐ATPsynthase (Faccenda & Campanella, 2012). During ischaemia, this enzyme runs in reverse, acting as an ATPase and leading to consumption of the cytosolic pool of ATP, in order to preserve the mitochondrial membrane potential (ΔΨ_m_; Rouslin, Broge, & Grupp, 1990; Vinogradov, 2000). The inhibitory protein ATPIF1 (IF_1_) is a nuclear encoded protein of 84 amino acids, which blocks the reversal of the F_1_F_o_‐ATPsynthase (ATPase activity), thus preventing the hydrolysis of ATP, at the expense of the ΔΨ_m_ (Campanella et al., 2008; Campanella, Parker, Tan, Hall, & Duchen, 2009). IF_1_, which is resident in the mitochondrial matrix (MM), is also correlated with correct mitochondrial morphology and programmed cell death (Faccenda et al., 2017; Faccenda, Tan, Duchen, et al., 2013; Faccenda, Tan, Seraphim, et al., 2013). The overexpression of IF_1_ is a common feature of many human cancers and has been proposed to be a pivotal mechanism to drive the nuclear retrograde response, ensuring metabolic adaptation, survival, and proliferation of cancer cells (Formentini, Sánchez‐Aragó, Sánchez‐Cenizo, & Cuezva, 2012). IF_1_ has also been proposed to facilitate mitophagy under ischaemic conditions. Mitophagy is an autophagic specialized process that allows the recycling of mitochondrial components (Campanella & Klionsky, 2013; Youle & Narendra, 2011). Thus, in response to oxygen reperfusion, IF_1_ overexpression promotes the recruitment of the ubiquitin ligase Parkin on mitochondrial membranes, allowing the execution of mitophagy (Matic et al., 2016). Parkin ubiquitinates proteins of the outer mitochondrial membrane (OMM), thus enabling their recognition by the ubiquitin‐binding protein p62/SQSTM1 (Sequestosome 1). This leads to the processing of defective mitochondria (Bjørkøy et al., 2005; Narendra & Youle, 2011) through recruitment of autophagosomes (East & Campanella, 2016). IF_1_ is therefore a preferred target to chemically intervene and selectively inhibit the reversal of the F_1_F_o_‐ATPsynthase (ATPase). The fine‐tuned pharmacological regulation of IF_1_ could indeed represent a way to facilitate mitochondrial network fitness by enacting targeted autophagy, providing another tool in the search for pharmacological regulators (Georgakopoulos, Wells, & Campanella, 2017). Following a chemoinformatic screening based on the structure of BMS199264, which was reported to selectively inhibit the F_1_F_o_‐ATPase activity and to exhibit cardioprotective activities (Grover & Malm, 2008), we found a new compound, BTB‐06584 (hereinafter referred to as BTB). The latter was able to maintain ATP levels during impairment of respiration and to operate in an IF_1_‐dependent manner to mediate protection in ischaemia/reperfusion assays (Ivanes et al., 2014).

Even though we succeeded to show a beneficial effect of the compound BTB also in vivo, by using the zebrafish mutant *pinotage* (*pnt* ^*tq209*^; Ivanes et al., 2014), some of the core chemical features such as (a) the presence of sulfonyl groups and (b) tendency to precipitate in solution were not compatible with use in vivo. We therefore refined the design of BTB to allow systemic implementation of this targeting strategy. We replaced the sulfonyl groups of the sulfide backbone of the BTB with sulfoximidoyl moiety, obtaining a “sulfoximine,” in which one oxygen and one nitrogen atom are connected to the central sulfur atom. Sulfoximines were discovered by Bentley and colleagues in the early 1950s (Bentley, McDermott, & Whitehead, 1950) and they are isoelectronic to sulfones, with a nitrogen atom that introduces an asymmetry of the central sulfur atom (Frings, Bolm, Blum, & Gnamm, 2017; Lücking, 2013, 2019). The interest in these formulations was initiated with the synthesis of *N*‐cyano‐sulfoximine (sulfoxaflor), which exhibited strong insecticidal properties (Zhu et al., 2011). More recently, other sulfoximines have been developed for potential medical applications such as Amgen's GKRP disruptor (1, AMG‐3969; Nishimura et al., 2014), Astra Zeneca's ATR inhibitor (AZD6738; Foote et al., 2018), and the PTEFb/CDK9 inhibitor atuveciclib (Bay 1143572; Lücking et al., 2017). The latter has entered clinical trials (Lücking et al., 2017), encouraging the use of similar compounds to investigate therapeutic strategies. A biologically active sulfoximine analogue of BTB could allow the pharmacological targeting of the F_1_F_o_‐ATPase in a wide range of conditions, thus making possible potential new therapeutic applications. Here, we present the chemical background and pharmacological validation of the newly synthesized NH‐sulfoximine derivative of BTB (NHS) and show that NHS could be as a possible co‐adjuvant to oncological chemotherapy by selectively blocking the consumption of ATP (ATPase activity) by mitochondria. NHS could also be used identify those malignancies in which the pathological homoeostasis is dependent on the F_1_F_o_‐ATPase.

## 2 METHODS

### 2.1 Plasmids and transfections

The down‐regulation of IF_1_ in the human neuroblastoma cell line SH‐SY5Y was achieved by using the pGIPZ shRNA vector V3LHS_409663 (target sequence: 5′‐ATGGTTCTGGTTTAACTAATA‐3′) TurboGFP (tGFP) tagged. All transfections were performed by using the Ca^2+^ phosphate method as previously demonstrated (Morelli et al., 2003). These down regutlated cells are referred to as SH‐IF_1_kd cells.

### 2.2 Cell lines

SH‐SY5Y *wild type*, SH‐SY5Y IF_1_kd and Hela cells were cultured in DMEM F‐12 medium (Corning) and DMEM (Thermo Fisher Scientific, 11965092), respectively, supplemented with 10% heat‐inactivated FBS (Corning 35‐015‐CV), 0.5% penicillin (Corning), 5% streptomycin (Corning) at 37°C under humidified conditions, and 5% CO_2_.

### 2.3 Treatments

Cells were treated with NHS (a gift from Prof. Dr Carsten Bolm, RWTH Aachen University) **2** at (1 μM) and/or etoposide (10 μM) and/or 2‐deoxyglucose (2‐DG; 10 mM) for 18 h. Etoposide binds to DNA and topoisomerase II enzyme causing DNA replication errors and inducing apoptosis. 2‐DG competitively inhibits the production of glucose‐6‐phosphate.

### 2.4 Western blotting

After 18‐h treatment, cells were washed in PBS (Thermo Fisher Scientific, 10010049) and collected by gentle scraping in ice‐cold RIPA buffer (Tris HCl pH 7.4 50 mM, NaCl 150 mM, EDTA 1 mM, MgCl_2_ 5 mM, 1% Triton and 0.1% SDS) to detect proteins. Protein concentration was estimated by using a BSA protein assay kit (BSA, AppliChem). Equal amounts of protein (40 μg) were mixed with a Laemmli sample buffer (Bio‐Rad, 161‐0747) and boiled at 95°C for 2 min. The samples were resolved by SDS‐PAGE (Serva), transferred to PVDF membranes (Merck Millipore, ABC821), and blocked in 5% non‐fat dry milk (AppliChem) in TBST (50 mM Tris, 150 mM NaCl, and 0.05% Tween 20, pH 7.5) for 1 h. Then, they were incubated with the appropriate diluted primary antibodies at 4°C overnight: anti‐p62 1:1000 (MBL, PM045), anti‐LC3 1 μg·ml^−1^ (Novus Biologicals, NB100‐2220), anti‐GAPDH 1:10,000 (Abcam, ab8245), anti‐α‐tubulin 1:1000 (Santa Cruz Biotechnology, sc‐8035), anti‐HSP90 1:1000 (Santa Cruz Biotechnology, sc‐69703,), anti‐IF_1_ 1 μg·ml^−1^ (Abcam, ab81061), anti‐AMBRA1 1 μg·ml^−1^ (Merck Millipore, ABC131), anti‐Parkin 1:1000 (Abcam, ab77924), anti‐PINK1 1:1000 (Abcam, ab23707), anti‐PARP 1:1000 (Santa Cruz Biotechnology, sc‐8007), anti‐caspase 3 1:1000 (Abcam, ab4051), ATP‐β antibody 1:1000 (Abcam, ab14730), β‐actin 1:1000 (Abcam, 1b8227), VDAC 1:1000 (Abcam, ab34726), or anti‐MTCO1 0.5 μg·ml^−1^ (Abcam, ab14705). Membranes were washed in TBST (3 × 10 min at room temperature) and then incubated with corresponding peroxidase‐conjugated secondary antibodies (Bio‐Rad) for 1 h at room temperature. After a further washing step (3 × 10 min at room temperature in TBST), proteins were detected by using chemiluminescence HRP substrate kit (Merck Millipore) via Amersham Imager 600. Immunoreactive bands were analysed by performing densitometry with ImageJ software. The immuno‐related procedures used comply with the recommendations made by the *British Journal of Pharmacology*.

### 2.5 Imaging mitochondrial membrane potential (ΔΨ_m_)

For the majority of the experiments, we used tetramethylrhodamine methyl ester (TMRM; 50 nM, Invitrogen, T668) in “redistribution mode” (Duchen, Surin, & Jacobson, 2003). The dye was allowed to equilibrate and was present continuously. TMRM distributes between cellular compartments in response to different potentials and is preferentially accumulated inside the mitochondrial matrix. At concentrations <50 nM, the fluorescent signal shows a simple relationship with the dye concentration (non‐quench mode) so that signal intensity is linearly correlated with mitochondrial potential. TMRM fluorescence intensity was quantified by removing all background signal by “thresholding” and measuring the mean TMRM fluorescence intensity in the pixels containing mitochondria. Thus, the signal is independent of mitochondrial mass and only reflects the dye concentration within individual mitochondrial structures. Mitochondrial depolarization is indicated by the redistribution of dye from mitochondria into the cytosol and can be induced by addition of 1 mM carbonyl cyanide 4‐(trifluoromethoxy) phenylhydazone (FCCP), which was performed at the end of every experiment as a control.

### 2.6 Luciferase measurements

Luminescence was measured in a custom‐built luminometer made in collaboration with Cairn Research (Faversham, Kent). Cells were perfused with Krebs–Ringer bicarbonate buffer, supplemented with 1 mM CaCl_2_ and 50 mM luciferin (Sigma‐Aldrich, L9504). The light emitted by a coverslip of transfected cells ranged from 1000 to 10,000 counts per second (cps) with backgrounds lower than 50 cps. All compounds employed in the experiments were tested for non‐specific effects on the luminescent signal—none were observed.

### 2.7 MgG assay

Changes in cellular ATP content were indirectly measured by using the cell permeant fluorescent dye MgG (Invitrogen, M3735). MgG fluorescence is a measure of concentration of free Mg^2+^, which has a strong affinity for ATP, whereas its affinity for ADP is 10 times less. Therefore, ATP hydrolysis is associated with a rise in free Mg^2+^ corresponding to an increase in fluorescence (Ivanes et al., 2014). After 18‐h treatment, cells were washed (3×) with HBSS (Thermo Fisher Scientific, 14175095) and were incubated with 5 μM MgG for 30 min at 37°C under humidified conditions and 5% CO_2_. After incubation and washing with HBSS (3×), cells were incubated in DMEM for 30 min at 37°C under humidified conditions and 5% CO_2_. Cover glasses were mounted within an Attofluor® metal cell chamber for microscopy and were imaged in HBSS. Continuous live imaging was carried out by using an Eclipse Ti inverted microscope (Nikon, Kingston upon Thames, UK) with an optoscan light source (Cairn, Faversham, UK) and a Leica electron multiplying CCD camera controlled through IQ2 software (Andor, Belfast, UK). The objective used was 40× CFI S FLUOR oil immersion. After 2 min of recording, 1 mM sodium cyanide (NaCN) and/or 2 mM iodoacetic acid (IAA) were added to inhibit all ATP‐generating pathways in order to measure ATP consumption. All samples were recording until they reached the plateau.

### 2.8 Cell viability assay

Cell viability was measured by using Crystal Violet (CV) that binds proteins and DNA proportionally to their quantity (Feoktistova, Geserick, & Leverkus, 2016). After 18‐h treatment, cells were fixed in 4% paraformaldehyde (AppliChem, A3813) in PBS 1× buffer and stained by using 0.1% CV for 20 min at room temperature. Samples were thoroughly washed with ddH_2_O and air‐dried. The remaining dye was dissolved by using 10% acetic acid solution (AppliChem, 181009). The absorbance (λ) was measured at 595 nm (Varioscan Lux, Thermo Fisher Scientific).

### 2.9 Cell cycle analysis

After 18‐h treatment, cells were harvested by using 0.05% trypsin (Corning, 25‐054‐CI), centrifuged (250g, 5′ at RT) and incubated with 70% ethanol for 20 min at −20°C. After that, 10 mg·ml^−1^ propidium iodide was added before analysis. Cells were scanned for DNA content by using a CytoFLEX flow cytometry (Beckman Coulter, USA). The proportion of cells in G0/G1, S G2/M, and sub G1 phase was quantified by using CytExpert software (Beckman Coulter). The software calculates the percentage of cells in each phase.

### 2.10 Analysis of mitochondrial Parkin recruitment

HeLa cells, grown on borosilicate cover glasses (Thermo Fisher Scientific), were co‐transfected with mCherry‐Parkin (Addgene, 23956) and either mitochondria‐targeted GFP or one of the GFP‐tagged IF_1_ clones (wild type, dominant negative E30A, or constitutively active H49P) as previously demonstrated (Faccenda et al., 2017). After 48 h from transfection, cells were fixed with 4% paraformaldehyde in PBS (10 min) and prepared for immunostaining. Briefly, cells were permeabilized in 0.1% Triton X‐100 in PBS for 15 min and incubated in blocking solution (10% normal goat serum, Thermo Fisher Scientific, PCN5000; 3% BSA, Sigma‐Aldrich, A2153; 0.01% Triton X‐100 in PBS) for 1 h. Cells were then incubated overnight with an anti‐ATPβ (Abcam, ab14730) 1:500 in blocking solution, washed 5 times in PBS, and incubated for 2 h with an Alexa Fluor 647‐conjugated anti‐Mouse IgG1 (Thermo Fisher Scientific, A21240) 1:500 in blocking solution. After a further washing (5 times in PBS), cells were mounted on slides using mounting medium with DAPI (Abcam, ab104139).

### 2.11 Mitochondrial ROS detection

After 18‐h treatment, cells were harvested by using 0.05% trypsin, centrifuged at 250g, 5′ at RT, and 5 μM MitoSOX™ Red (Thermo Fisher Scientific, M36008) was added before analysis. Cells were analysed for mitochondrial ROS content by using a CytoFLEX flow cytometry and CytExpert 2.0 software (Beckman Coulter). MitoSOX™ Red probe exhibits red fluorescence when oxidized by mitochondrial‐derived superoxide.

### 2.12 Data and statistical analysis

The data and statistical analysis comply with the recommendations of the *British Journal of Pharmacology* on experimental design and analysis in pharmacology (Curtis et al., 2018). Blinding and/or randomization methods were not required as the study was solely developed in vitro. The results are presented as mean ± SEM. The number of independent experiments was 5 (*n* = 5), and statistical analysis was carried out using these independent values. Student's *t*‐test and ordinary two‐way ANOVA followed by Holm–Sidak test were used for cellular correlation and multiple comparisons, respectively. The correlation between Parkin and mitochondria (ATPβ) was analysed by linear regression and calculated by Pearson correlation coefficient. Statistical significance was accepted at *P* < 0.05. Post hoc tests were carried out only if *F* achieved *P* < 0.05. Statistical analysis was performed by using the GraphPad Prism 6 software.

### 2.13 Materials

All materials were purchased from Sigma‐Aldrich unless stated otherwise. 2‐DG, etoposide, sodium cyanide, IAA, Crystal Violet and propidium iodide were obtained from Merck Millipore; shRNA vector V3LHS_409663 from Qiagen; Magnesium Green™‐AM (MgG) from Invitrogen (M3735); and MitoSOX™ Red from Thermo Fisher Scientific (M36008).

### 2.14 Nomenclature of targets and ligands

Key protein targets and ligands in this article are hyperlinked to corresponding entries in https://www.guidetopharmacology.org and are permanently archived in the Concise Guide to PHARMACOLOGY 2019/20 (Alexander, Fabbro, et al., 2019; Alexander, Kelly et al., 2019).

## 3 RESULTS

### 3.1 Synthesis of NHS, the sulfoximine‐based analogue of BTB

**FIGURE 1:**
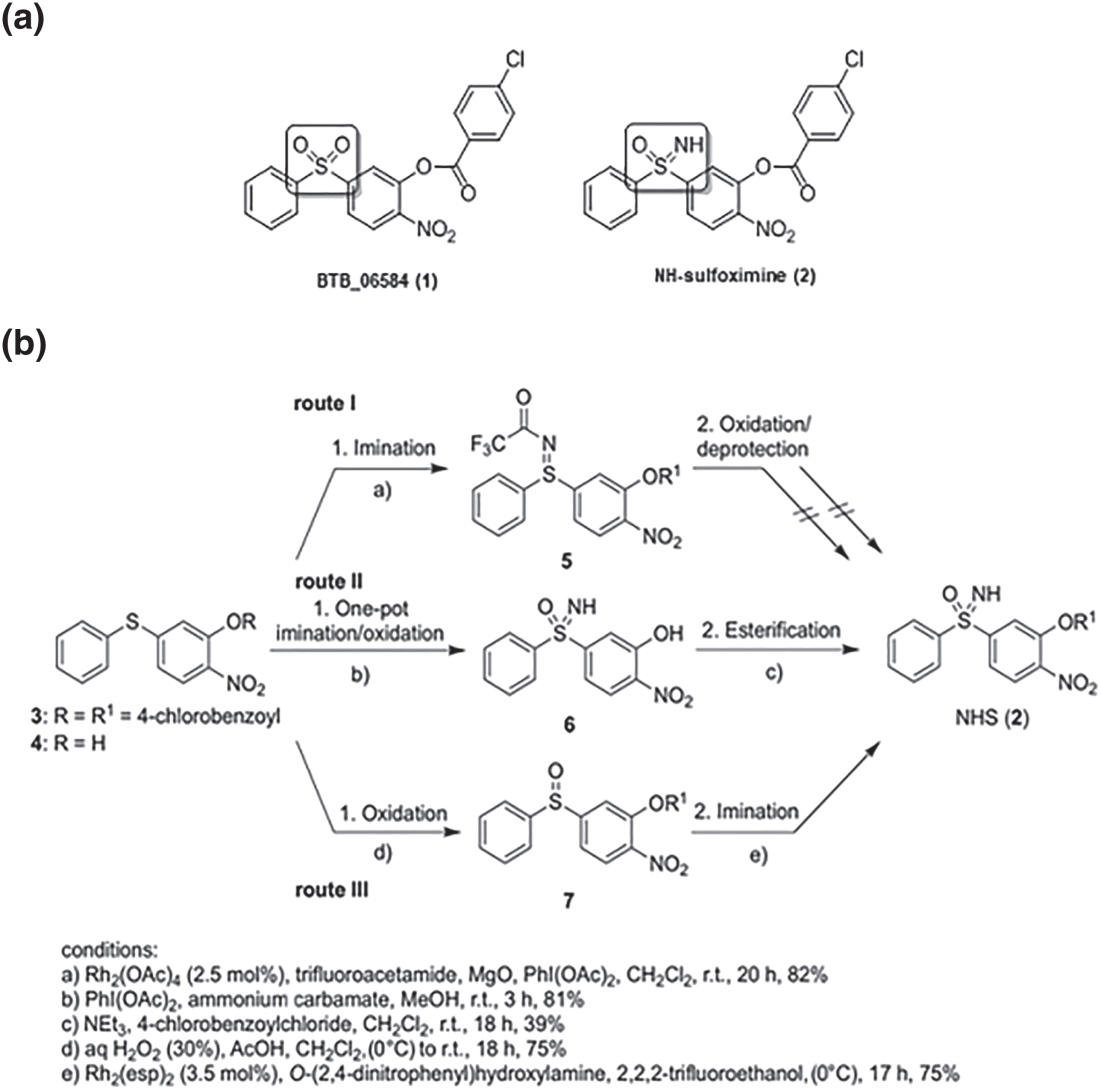
Synthesis of NHS as a sulfoximine analogue of BTB06584. (a) Structures of BTB06584 (**1**) and NHS (**2**). (b) Strategies followed to reach the final product are detailed in the figure in which conditions, yield of products, intermediates, and catalysts are also reported

Initially, three synthetic pathways to synthesize NHS (**2**) from sulfides (**3** or **4**) were considered. Those are shown in Figure 1 and briefly described here. Route I involved imination of sulfide (**3**; condition a) followed by oxidation and deprotection to give NHS (**2**) via sulfilimine (**5**). In route III, the sulfoximine stage was achieved by oxidation (condition d) of sulfide (**3**) to sulfoxide (**7**) and subsequent imination (condition e; for experimental details and analytical data of compounds prepared in routes I and III). Route II involved instead a shorter one‐pot imination/oxidation (condition b) of sulfide (**4**) followed by esterification (condition c). The initial focus on route I, in which sulfilimine (**5**) was synthesized in 82% yield by using a rhodium‐catalysed imination method with trifluoroacetamide (condition a; Okamura & Bolm, 2004), failed to oxidize sulfilimine (**5**), which prompted us to follow synthetic route III. Oxidation of sulfide (**3**) by in situ generated peracetic acid led to sulfoxide (**7**) in 75% yield (condition d; Golchoubian & Hosseinpoor, 2007). Considering the lability of the 4‐chlorobenzoyl ester towards basic *N*‐deprotection conditions, we decided to directly synthesize the NHS (**2**) from the sulfoxide (**7**). Fortunately, rhodium‐catalysed imination, employing *O*‐(2,4‐dinitrophenyl)hydroxylamine as the nitrogen source (Miao, Richards, & Ge, 2014), provided the desired NHS (**2**) in 75% yield (condition e). Following route II, a one‐pot imination/oxidation protocol was used to prepare the NH‐free sulfoximine (**6**) in 81% yield starting from sulfide (**4**; condition b; Tota et al., 2016) and synthesis of the 4‐chlorobenzoyl ester in the last step (Lee, Kim, Jun, Kim, & Lee, 2011) to obtain NHS (**2**) in 39% yield (condition e; Figure 1b). Details of the synthesis and physicochemical data of NHS are available in the Supplementary files.

### 3.2 Effect of NHS on ATP consumption

NHS (**2**) was initially evaluated in the NCI 60 Cell One‐Dose Screen (single high dose 1 μM), which reported growth inhibition in several cell lines (Figure S1).

**FIGURE 2:**
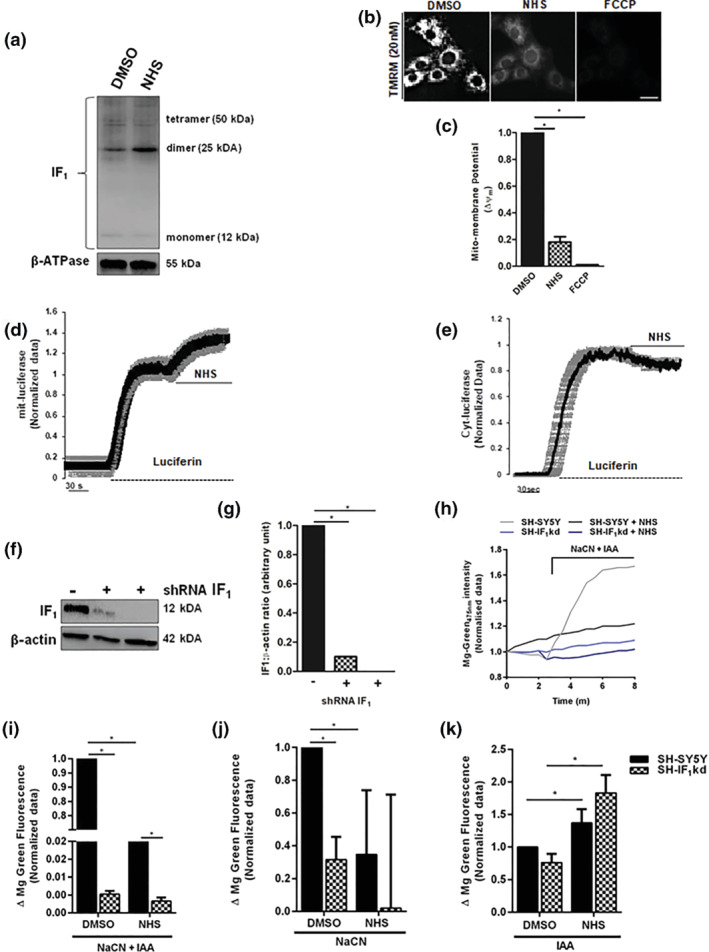
Effect of NHS on ATP dynamics. (a) NHS induces dimerization of IF_1_ (representative of *n* = 6). (b) ΔΨ_m_ assessment in SH‐SY5Y cells exposed to NHS (1 μM) via TMRM (20 nM) fluorescence. (c) Bar graph quantifying mito‐membrane potential (ΔΨ_m_). Data are shown as mean values ± SEM (*n* = 14). *P < 0.05, significantly different as indicated. (d–e) Representative traces as recorded by a luminometer in SH‐SY5Y cells transfected with mitochondria‐targeted (mit) and citoplasmic‐targeted (cyt) luciferase and perfused with luciferin (100 μM). Once at plateau, cells were challenged with NHS (1 μM), and the kinetics monitored (*n* = 9). (f–g) SH‐SY5Y cells were stably transfected with pGIPZ GFP‐labelled vector (as described in Section 2), and IF_1_ down‐regulation was confirmed by western blot analysis in (f). (g) The bars show the change in IF_1_ expression, normalized to β‐actin levels and expressed as mean value ± SEM (*n* = 9). *P < 0.05, significantly different as indicated. (H) Representative traces of the changes in MgG fluorescence in response to NaCN and IAA treatment. (I) The bars show the change of MgG fluorescence corresponding to ATP depletion after 18‐h treatment with NHS 1 μM in the presence of both NaCN (1 mM) and IAA (2 mM). Data are normalized to the untreated cells and expressed as mean value ± SEM (*n* = 11). *P < 0.05,significantly different as indicated. (j and k) The increase of MgG fluorescence was then assessed in response to (j) NaCN or (k) IAA after treatment with NHS 1 μM. Data are normalized to the untreated cells and expressed as mean values ± SEM (*n* = 15). *P < 0.05, significantly different as indicated

In order to confirm that this effect was due to inhibition of the mitochondrial F_1_F_o_‐ATPase, we tested whether, in SH‐SY5Y cells, IF_1_ was effectively activated by NHS. Figure 2a shows the increased degree of IF_1_ dimerization following NHS treatment, which led to the collapse of ΔΨ_m_ (Figure 2b,c). This implied that the arrest of cellular growth in cancer cells could be due to alterations in the bioenergetic equilibrium of those cells relying on the mitochondrial F_1_F_o_‐ATPase. We therefore performed a series of assays to map the levels of ATP (Ivanes et al., 2014) in SH‐SY5Y cells by assaying luciferase targeted to either mitochondria or the cytosol (Figure 2d,e), which confirmed that NHS modifies ATP fluxes in the two compartments as previously recorded for BTB (Matic et al., 2016). In both wild‐type cells and stably down‐regulated for IF_1_ (SH‐IF_1_kd previously obtained by Matic et al., 2016; Figure 2f,g), we measured the level of free intracellular Mg^2+^ with the fluorescent dye MgG (5 μM)—as a read‐out of ATP dissipation—in response to chemical inhibition of cellular respiration. Intracellular Mg^2+^ levels can be measured to indirectly assess free ATP levels and therefore infer the rate of ATP consumption by mitochondria (Ivanes et al., 2014). In response to chemical ischaemia induced by co‐treatment with 1 mM NaCN and 2 mM IAA, which inhibit cytochrome oxidase (complex IV) and glycolysis, respectively, we observed a significant reduction in the release of free Mg^2+^ in SH‐SY5Y cells treated with NHS in comparison to vehicle treatment (DMSO) and to SH‐IF_1_kd in both DMSO and NHS treatment (Figure 2h,i).

To further investigate the target of the NHS‐dependent inhibition of ATP hydrolysis, we used NaCN and IAA separately. Figure 2j,k shows that NHS successfully prevents ATP consumption when OXPHOS is impaired, while inhibition of glycolysis further accelerates the accumulation of free Mg^2+^.

### 3.3 Effect of NHS on cellular viability and apoptosis

**FIGURE 3:**
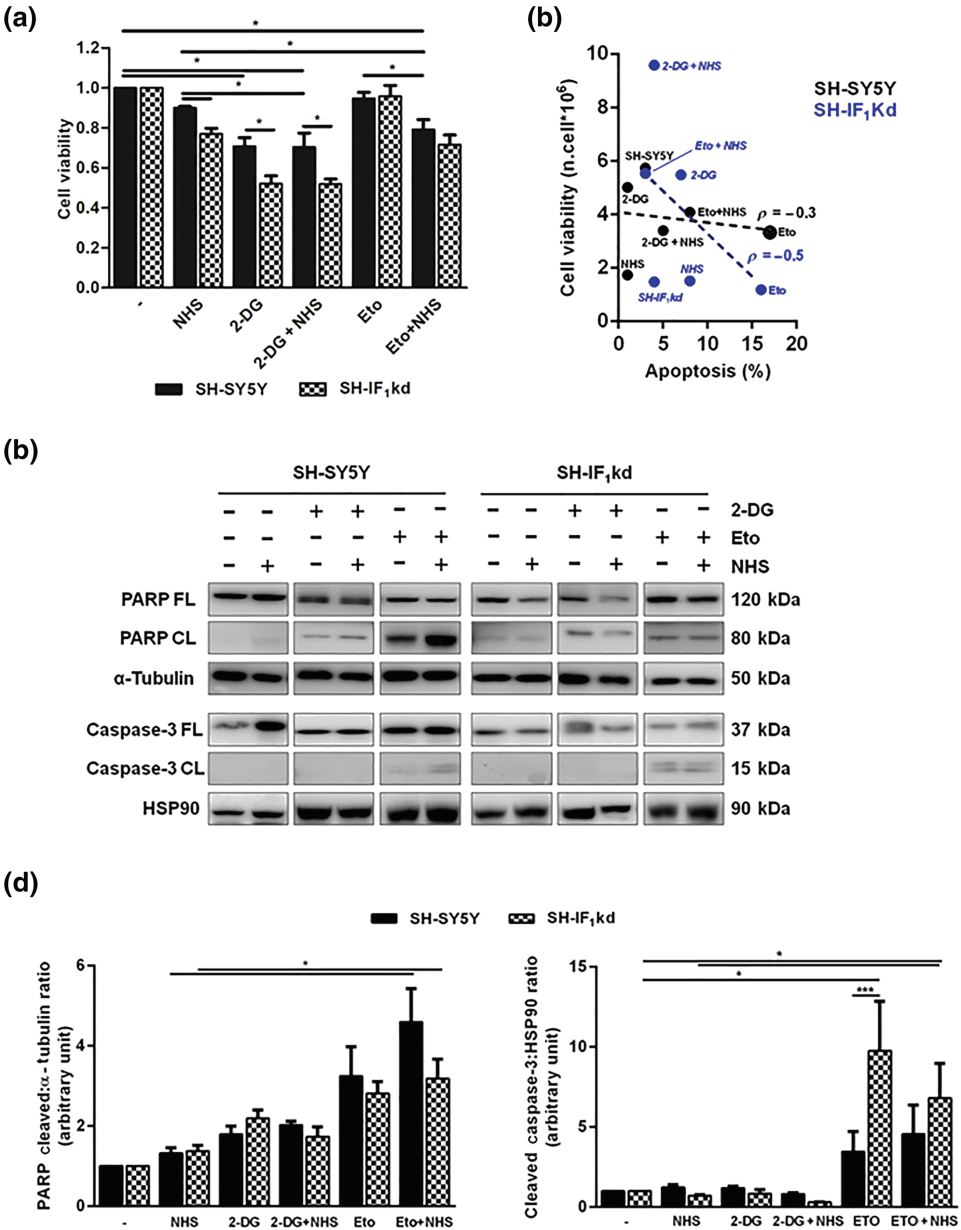
Effect of NHS on cellular viability and apoptosis. (a) The graph shows the change in cell viability, via Crystal Violet assay, of SH‐SY5Y and SH‐IF_1_kd after 18‐h treatment with NHS 1 μM and/or 2‐DG 10 mM and/or etoposide (Eto; 10 μM). Data are normalized to the untreated cells and expressed as mean values ± SEM (*n* > 9). *P < 0.05, significantly different as indicated. (b) Scatter plot of apoptosis versus cell viability assay in IF_1_ positive and IF_1_kd cells quantified via Pearson's correlation coefficient (*ρ* = −0.3 SH‐SY5Y; *ρ* = −0.5 SH‐IF_1_kd). Apoptosis was measured by propidium iodide staining and flow cytometry (*n* = 5). Cell viability was detected via Trypan blue assay (*n* = 5). (c) Representative western blotting of SH‐SY5Y and SH‐IF_1_kd cells exposed to drugs (18 h). The blot shows the change in the amount of full length (FL) and cleaved (CL) isoform of PARP and caspase‐3 normalized to α‐tubulin and HSP90, respectively, and quantified in (d). Data are expressed as mean values ± SEM (*n* = 6). *P < 0.05, significantly different as indicated

Subsequently, the effects of NHS treatment on cell viability (18 h) were analysed, and a significant decrease was observed in both lines, but mainly in SH‐IF_1_kd cells (Figure 3a). The effect on cell viability was greater when NHS was combined with the DNA targeting agent etoposide or the competitive inhibitor of hexokinase (the enzyme catalysing the first step of glycolysis, consisting of glucose phosphorylation at the expense of ATP), 2‐DG. The latter successfully blocked glycolysis per se, inducing a sharp decrease in the survival rate of SH‐SY5Y cells, in which the lack of IF_1_ further reduces the cell survival.

By using flow cytometry, we mapped the specific steps of the cell cycle after NHS treatment (Table 1). In SH‐SY5Y cells treated with 2‐DG, the assay reported an increase in the G0/G1, which was accompanied by a containment of the G2 phase. This is a characteristic feature of apoptosis, which was not retained in cells treated with etoposide, which instead increased G2 phase. Nonetheless, NHS failed to prolong the G2 phase of the cell cycle following 2‐DG treatment, which made us conclude that there was no clear correlation between loss of cellular viability and apoptosis (Figure 3b). In order to corroborate this further, we analysed, by western blotting, the proteins involved in the signalling and execution of the intrinsic pathway of apoptosis. Expression and cleavage of PARP and caspase‐3 were therefore assessed after the various conditions of treatment (Figure 3c,d). Even though the sole addition of NHS did not show any effects on both PARP and caspase‐3 cleavage, it increased the effect driven by etoposide in wild‐type SH‐SY5Y cells (Figure 3c,d). On the contrary, 2‐DG, either alone or in combination with NHS, had weak effects on PARP cleavage, parallel to no effect on caspase‐3 cleavage, thus highlighting that its effects on cell viability was solely mediated by a metabolic impairment of the underlying glycolysis that takes place in cancer cells (Figure 3c,d).

**TABLE 1:** Cell cycle analysis

SH‐SY5Y	G0G1, %	S, %	G2, %	Sub G1, %
CTR	37.83 ± 12.01	20.73 ± 6.2	33.33 ± 7.65	3.82 ± 1.6
NHS	37.39 ± 11.03	27.21 ± 9.14	39.40 ± 8.88	1.37 ± 0.5
2‐DG	33.19 ± 12.12	22.63 ± 7.33	40.55 ± 7.66	1.43 ± 0.6
2‐DG + NHS	46.68 ± 5.41^†^	20.25 ± 3.19	24.43 ± 5.56^†^	6.15 ± 2.1
ETO	36.65 ± 5.48	20.51 ± 12.5	35.01 ± 8.89	4.58 ± 1.4
ETO + NHS	41.45 ± 4.97	18.58 ± 5.39	23.57 ± 3.65	3.45 ± 1.2
SH‐IF_1_kd				
CTR	33.84 ± 16.01	19.42 ± 5.35	39.44 ± 6.9	8.69 ± 2.1
NHS	33.19 ± 10.55	24.60 ± 1.2	40.55 ± 3.56	1.43 ± 0.7
2‐DG	34.23 ± 7.67	21.76 ± 1.36	42.78 ± 9.5	5.55 ± 1.1
2‐DG + NHS	39.45 ± 15.66	18.90 ± 5.78	37.06 ± 10.1	3.44 ± 1.5
ETO	32.39 ± 17.43	21.38 ± 10.14	45.63 ± 2.56*	1.89 ± 0.6
ETO + NHS	35.60 ± 11.62	21.40 ± 4.88	41.79 ± 9.5	3.75 ± 1.5

*Note*: The table shows the change in the percentage of PI positive cells after 18‐h treatment with drugs in SH‐SY5Y and SH‐IF_1_kd cells. Data are normalized to the untreated cells and expressed as mean value ± SEM.

* *P* < 0.05, significantly different from control (CTR).

† *P* < 0.05, significantly different from 2‐DG alone (*n* > 3).

### 3.4 Modulation of autophagy by NHS treatment

**FIGURE 4:**
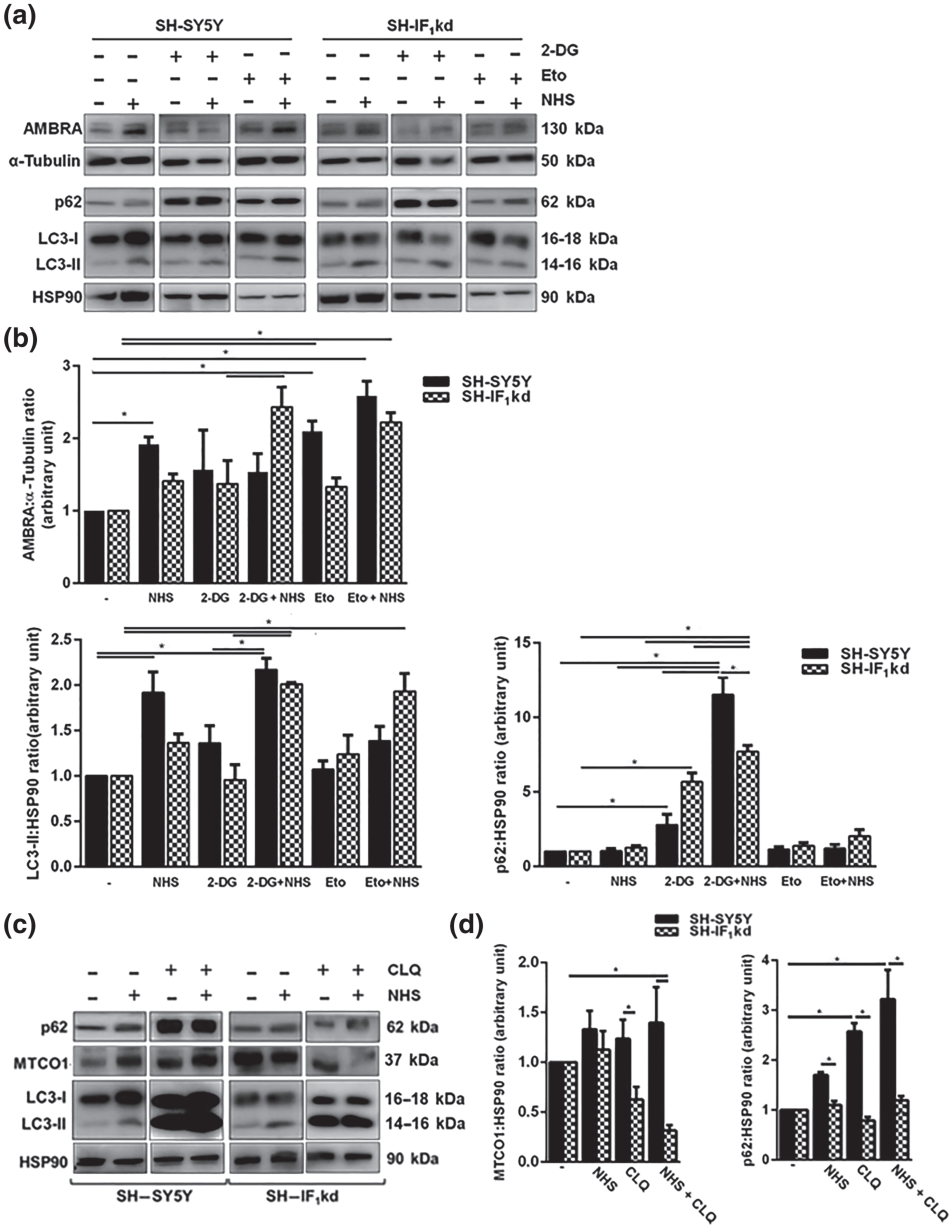
Autophagy modulation after NHS treatment. (a) Representative western blotting of the autophagic markers AMBRA, p62, and LC3‐I/LC3‐II ratio in cells exposed to treatments with NHS 1 μM and/or 2‐DG 10 mM and/or etoposide (Eto; 10 μM) in both SH‐SY5Y and SH‐IF_1_kd cells for 18 h. (b) The bars show the change in AMBRA, p62, and LC3‐II amount normalized to α‐tubulin and HSP90 levels, respectively, and expressed as mean value ± SEM (*n* = 5). *P < 0.05, significantly different as indicated. (c) Representative western blot of LC3, p62, ATPβ, and MTCO1 in IF_1_ and IF_1_kd cells pretreated with chloroquine (CLQ, 1:1000) in combination with NHS 1 μM. (d) The bars show the change in MTCO‐1 amount normalized to HSP90 levels. Data are expressed as mean values ± SEM (*n* = 5). *P < 0.05, significantly different as indicated

Considering that NHS alone does not alter apoptosis despite its effect on cellular viability, we evaluated if this was due to an engagement of autophagy. The reduction in the ATP levels recorded following inhibition of the F_1_F_o_‐ATPase could well activate this process for energy preservation. By western blotting analysis of both SH‐SY5Y and SH‐IF_1_kd cells exposed to NHS (1 μM for 18 h), we observed that the expression level of the autophagy regulator AMBRA‐1 was actually increased, and LC3 cleaved and lipidated to form LC3‐II (Figure 4a,b). This was independent of the IF_1_ expression level or co‐treatment with either 2‐DG or etoposide. Actually, 2‐DG treatment had an effect on LC3 lipidation (LC3‐II) but not on AMBRA‐1, thus implying that NHS might operate upstream (Figure 4a,b). NHS was then tested against the autophagic flux inhibitor chloroquine, which did not modify its effects except when the two were combined in SH‐IF_1_kd cells (Figure 4c,d). In these cells, stabilization of p62 was greater, associated with the degradation of the mitochondrially encoded cytochrome c oxidase I, MTCO1 (used as a read‐out for mitophagy; Figure 4c,d).

**FIGURE 5:**
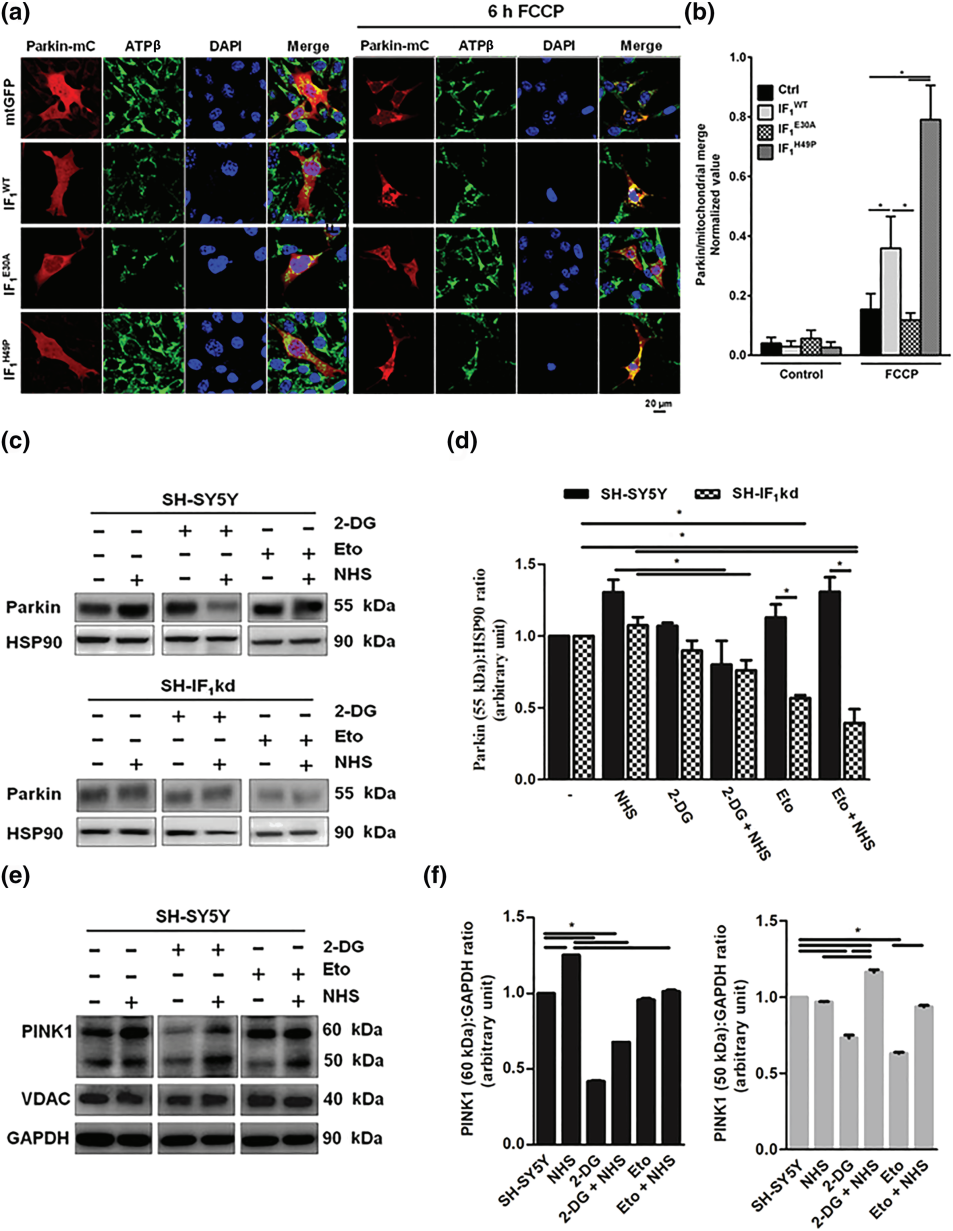
Pattern of mitochondrial Parkin translocation during mitophagy activation according to IF_1_ oligomerization. (a) Representative IF of HeLa cells co‐transfected with Parkin‐mCherry and either a control plasmid or one of the IF_1_ clones. Then, cells were treated for 6 h with DMSO and FCCP, fixed, and immunostained for ATPβ (green), Parkin (red), and DAPI (blue). (b) Co‐localization was quantified by using Pearson's correlation coefficient. *P < 0.05, significantly different as indicated (*n* = 7). Representative western blotting of (c) Parkin and (e) PINK1 and VDAC expression in SH‐SY5Y and SH‐IF_1_kd cells treated with NHS 1 μM and/or 2‐DG 10 mM and/or etoposide (Eto; 10 μM) for 18 h. They were normalized on the basis of HSP90 and GAPDH levels, respectively. Parkin and PINK1 expressions were respectively quantified in (d) and (f). Data are shown as mean values ± SEM (*n* = 5). *P < 0.05, significantly different as indicated

IF_1_ facilitates mitophagy by (a) collapsing the ΔΨ_m_, (b) stabilizing PINK‐1, and (c) promoting PARK2 translocation in mitochondria (Lefebvre et al., 2013). The data reported in Figure 5a,b show that FCCP‐induced mitochondrial accumulation of PARK2 is indeed high in those cells, which express fully functional IF_1_ and impaired in those bearing the mutant isoforms of the protein. NHS must therefore act differently from IF_1_ in the regulation of mitophagy as its addition even though collapses the ΔΨ_m_ (see Figure 2c,d) did not affect the level of PARK2 (Figure 5c,d) or processing of PINK‐1, which remained unchanged (Figure 5e,f).

The variations in both PARK2 and PINK‐1 recorded with 2‐DG and etoposide (Figure 5c–f) indicate that NHS does not engage mitochondrial autophagy during the execution of cell death.

**FIGURE 6:**
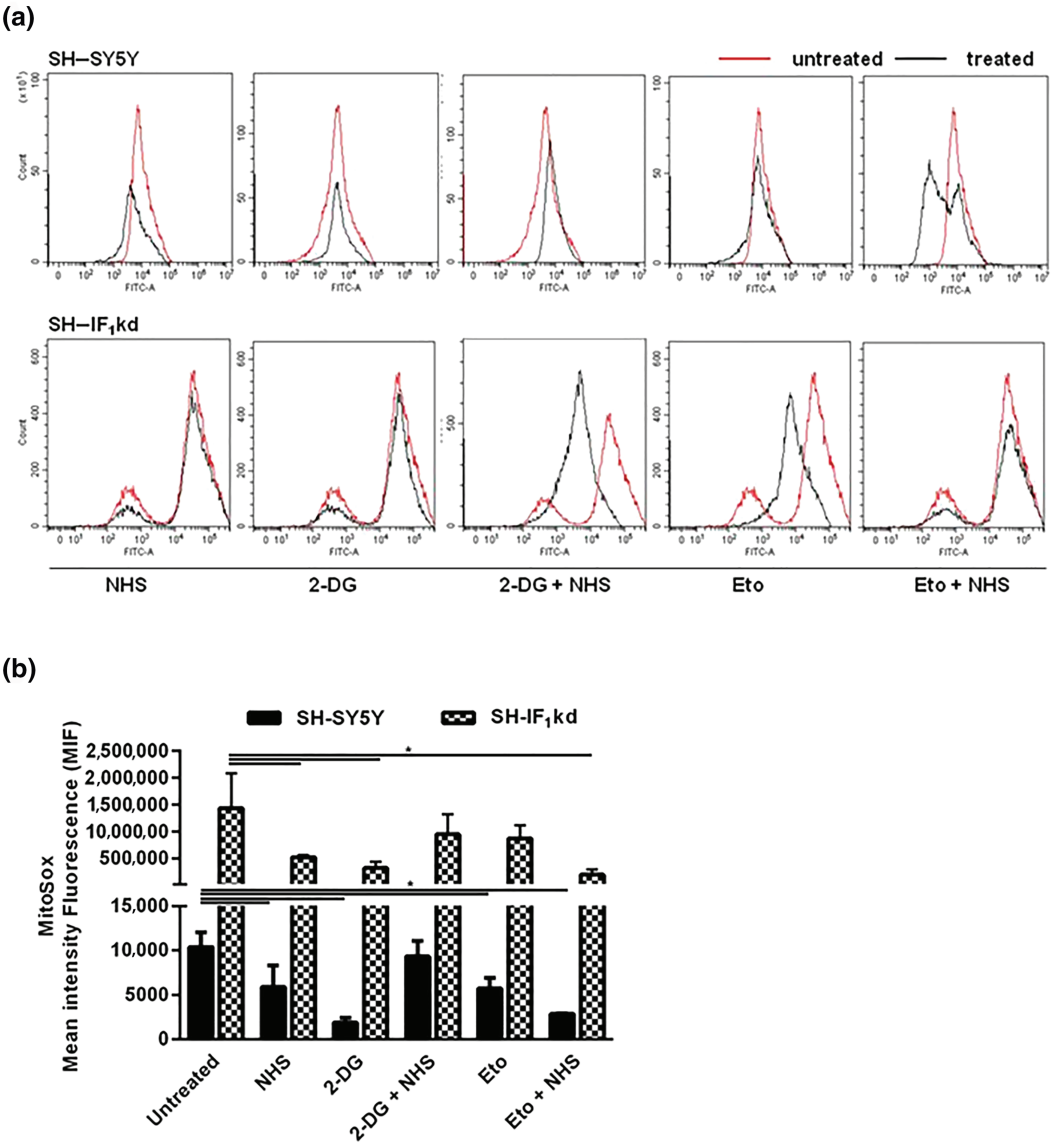
NHS effect on the regulation of mitochondrial ROS production. (a) A representative flow cytometric histogram of MitoSOX^TM^ Red fluorescence positive cells at 18 h after NHS 1 μM and/or 2‐DG 10 mM and/or Eto 10 μM in both SH‐SY5Y and SH‐IF_1_kd cells. (b) The graph shows the change in mean intensity fluorescence (MIF) after treatments. Data are normalized to the untreated cells and expressed as means ± SEM (*n* = 5). *P < 0.05, significantly different as indicated

The selectivity of action on mitochondrial function by NHS was further tested by mapping the accumulation of mitochondrial ROS production (superoxide) with use of MitoSOX^TM^ Red. As shown in the raw data reported in Figure 6a and quantified in Figure 6b, NHS statistically reduced the production of mitochondrial ROS in both SH‐SY5Y wild type and SH‐IF_1_kd cells. Interestingly, also for this parameter, the effect was greater when IF_1_ was depressed (in SH‐IF_1_kd cells) and in combination with etoposide and 2‐DG. These results confirmed the action of the compound is due to the targeting of the F_1_F_o_‐ATPase.

**FIGURE 7:**
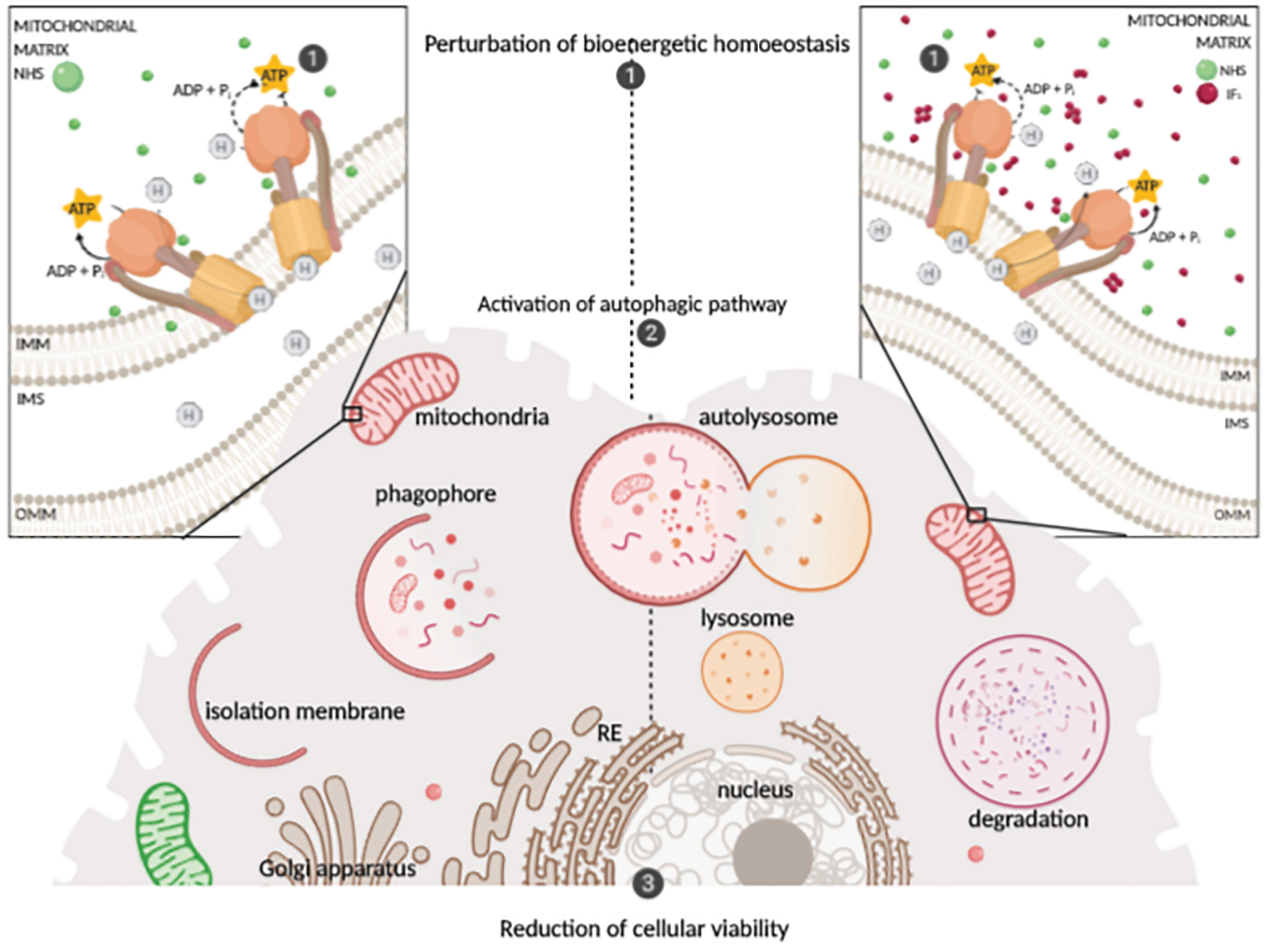
Working model for the NHS‐mediated imbalance of the pathological metabolism in cancer cells, leading to an arrest of proliferation. H, hydrogen; IMS, intermembrane space; IMM, inner mitochondrial membrane; OMM, outer mitochondrial membrane

## 4 DISCUSSION

The increased understanding of mitochondrial bioenergetics has not been associated with the development of pharmacological tools to selectively counteract mitochondrial alterations and, therefore, restore its homoeostatic function. The most obvious target to achieve this would be the complex V, which reverses the synthesis of ATP to its hydrolysis during conditions of reduced ΔΨ_m_ (see Campanella et al., 2009). The detrimental activity of the enzyme can be pharmacologically blocked by oligomycin or by inhibiting the electron transport chain complexes that fuel its function or the enzyme directly. Pharmacological tools that solely target the F_1_F_o_‐ATPase differentiating the consumption from the synthesis of ATP have not been yet developed with those parameters of safety needed for medical applications. Succeeding in this would represent an advance in molecular pharmacology and open the way to new therapeutic applications to treat those conditions in which the reversal of the F_1_F_o_‐ATPsynthase plays an active pro‐pathological role (Chinopoulos & Adam‐Vizi, 2010).

Over the years, the interest in such *selective* regulation of the F_1_F_o_‐ATPsynthase has concentrated our attention on the physiology and pharmacology of IF_1_, a protein that, in response to the inhibition of respiration, prevents the consumption of ATP by forcing the F_1_F_o_‐ATPase into an inhibited state (Boreikaite, Wicky, Watt, Clarke, & Walker, 2019; Faccenda & Campanella, 2012; Rouslin & Broge, 1996). In addition to the work generated following genetic modulation of IF_1_, we characterized a chemical compound, BTB, which selectively limits ATP consumption by the F_1_F_o_‐ATPsynthase by increasing the activation of IF_1_. The unfavourable chemical features of BTB have led to the modification of this molecule in order to improve the applicability of the compound and allow its use in translational research. This is the goal for which the sulfoximine analogue NHS was designed. Sulfoximines, Sulfonamides, and sulfonimidamides are promising, but not yet fully explored, variants of the more common sulfone or sulfonamide motif, which have tangible chemical advantages. The sulfoximine moiety provides chemical stability, often leading to favourable aqueous solubility and membrane permeability (Frings et al., 2017; Figure 1), which are desirable features for a molecule designed to target mitochondrial enzymes. Moreover, the nitrogen enables simple chemical modifications, which facilitate fine‐tuning of the molecular properties, making them a valuable addition to the medicinal chemistry tool box. With this background, NHS was synthesised and tested for its capacity to preserve ATP levels which would otherwise be consumed by mitochondria, during ischaemia‐mimicking conditions.

Even though deletion of IF_1_ damages the homoeostasis of cellular ATP leading to cell energy depletion, as revealed earlier (Faccenda et al., 2017; Fujikawa, Imamura, Nakamura, & Yoshida, 2012), NHS is still able to deliver a protective effect, which provides a tangible advantage over BTB and BMS, which operate solely in the presence of IF_1_. NHS decreases the viability of SH‐SY5Y cells, and its combination with 2‐DG blocks their glycolytic capacity. Such evidence suggests that the basal metabolism of the cell line is likely to be due to the reversal of the F_1_F_o_‐ATPsynthase (F_1_F_o_‐ATPase activity), which is necessary to retain the balance of metabolites essential for the cellular growth. Therefore, the inhibition of F_1_F_o_‐ATPase via NHS treatment affects the viability of cancerous cells. Interestingly, only a specific subset of cancer cells is affected by NHS treatment, implying a particular metabolism, which is therefore exploited to reduce viability (Figure S1). Such a mechanism was supported by the lack of activation of caspases and PARP (Figure 3d,e), which were engaged by the apoptotic stimulus etoposide, used as a positive control. The way in which NHS slows the viability of cancer cells could therefore operate via the perturbation of cellular bioenergetic homoeostasis, which involves adaptive processes, such as autophagy, which are also able to promote cell death (Singh et al., 2018).

This finding has clinical relevance and promise as it could form the basis of therapeutic protocols, providing greater selectivity of action (and hence safety), which are based on the exploitation of a feature unique to cancer cells, thus shielding the normal, non‐cancer cells.

The perturbation of the bioenergetic homoeostasis in SH‐SY5Y cells was paralleled by the activation of autophagy, as indicated by LC3 lipidation and AMBRA‐1 induction (Figure 4). The activation of autophagy reflects the dis‐equilibrium caused by the inhibition of F_1_F_o_‐ATPase by NHS, which delivered a similar effect even in the absence of IF_1_. In the SH‐IF_1_kd cells, an underlying autophagy was also present, which is indicative of the relevance of the F_1_F_o_‐ATPase in the cellular bioenergetics of SH‐SY5Y cells (Figure 4a,b).

The lack of IF_1_ capitalizes on the activation of macro‐autophagy by NHS, leading to a reduction of the overall content of mitochondrial proteins, such as MTCO1 and ATPβ, which are protected when IF_1_ is present (Figure 4c,d). This is apparently in contrast with the finding that both the expression of IF_1_ and its correct binding to the F_1_F_o_‐ATPase are required for the correct execution of Parkin‐mediated mitophagy, when the process is triggered by collapse of the ΔΨ_m_ (Figure 5). We therefore speculate that the stable inhibition of IF_1_ expression primes the targeting by the autophagosomes of a pool of mitochondria, which are able to withstand the segregation. The pharmacological challenge provided by NHS unlocks the process, and hence, the level of the MTCO1 (mtDNA‐encoded complex) decreases. NHS, by impairing the equilibrium, leads to a robust activation of the whole process. Because the chemical inhibition of the autophagic flux tangibly precipitates the process, we are also proposing that the anti‐mitophagy features gained by cells lacking IF_1_ are a consequence of the exploitation of this pathway (Figure 4c,d). The modulation of the respiratory by‐products recorded in response to NHS treatment demonstrates the effect of the compound on the bioenergetics of mitochondria (Figure 6). 537380399

IF_1_ holds the ability to reduce the accumulation of redox stress, but NHS is unable to mimic this. In the absence of IF_1_, NHS also increases the dynamics of ROS accumulation. The increased ROS levels caused by NHS are even higher in combination with 2‐DG, and this effect is, very likely, a consequence of impairment in the re‐equilibrium between ATP and ADP as a consequence of decreased glycolysis.

In conclusion, NHS is a chemical improvement on BTB and exposes the altered bioenergetics of highly glycolytic, rapidly proliferating cells, leading to an arrest of their growth. It represents therefore a valuable tool to selectively target pools of degenerated cells in which malignancy is supported by faulty mitochondria operating as consumers rather than producers of ATP. What is fascinating is that these cells are primed to undergo aggressive mitophagy, which is counteracted by the hyperactivation of IF_1_. The deletion of IF_1_, even though it leaves the F_1_F_o_‐ATPase exposed and hence open to targeting by NHS, drives cells far more readily to death.

## AUTHOR CONTRIBUTIONS

M.C. designed the study, analysed the data, and edited the manuscript; D.S., R. P., and O.C. conducted the experimental work and run their analysis; D.S. wrote the manuscript and designed the figures; A.M. conducted the experiments on cell cycle analysed with the support of C.M.; D.S., D.F., and R.A. designed, conducted, and analysed mitophagy; C.M.M.H., S.W., and C.B. took charge of the chemical synthesis and first validation on cancer cells of the NHS; All authors approved the final version of the manuscript.

## CONFLICT OF INTEREST

The authors declare no conflicts of interest.

## DECLARATION OF TRANSPARENCY AND SCIENTIFIC RIGOUR

This Declaration acknowledges that this paper adheres to the principles for transparent reporting and scientific rigour of preclinical research as stated in the *BJP* guidelines for Design & Analysis and Immunoblotting and Immunochemistry, and as recommended by funding agencies, publishers and other organisations engaged with supporting research.

## Supporting information


**FIGURE S1.** NCI 60 cell one‐dose screen.

## Data Availability

The authors confirm that the data supporting the findings of this study are available within the article and its supplementary materials.
