# Supplementary material for: NH‐sulfoximine: A novel pharmacological inhibitor of the mitochondrial F1Fo‐ATPase, which suppresses viability of cancerous cells
Source: Br J Pharmacol. 2020 Dec 14;178(2):298–311. doi: 10.1111/bph.15279 (PMC9328437; doi:10.1111/bph.15279)
Supplement: Supplementary file 1 — FIGURE S1. NCI 60 cell one‐dose screen. [file BPH-178-298-s001.docx]

### Details of chemical synthesis of NHS with physicochemical data

##### 2-Nitro-5-(phenylsulfonimidoyl)phenyl 4-chlorobenzoate (2)

A flame-dried and argon-flushed Schlenk tube was charged with Rh_2_(esp)_2_ (6.6 mg, 3.5 mol%), *O*-(2,4-dinitrophenyl)hydroxylamine (149 mg, 0.75 mmol) and 2,2,2-trifluoroethanol (2.5 mL).^1^ After cooling to 0 °C, 2‑nitro-5-(phenylsulfinyl)phenyl 4‑chlorobenzoate (**7**) (100 mg, 0.25 mmol) was added and the reaction mixture was stirred at 0 °C for 17 h. Water (5 mL) was added, and the reaction mixture was extracted with CH_2_Cl_2_ (4 x 10 mL). The combined organic layers were dried over anhydrous magnesium sulfate and the solvents were removed under reduced pressure. After purification by column chromatography (AcOEt/*n*-pentane 5:1 to 2:1) the product was obtained as a white solid (78 mg, 75%). Mp.: 57 – 59 °C.

^1^H NMR (400 MHz, CDCl_3_): *δ* = 8.16 (d, *J* = 8.6 Hz, 1H), 8.09 – 8.03 (m, 6H), 7.61 – 7.57 (m, 1H), 7.55 – 7.48 (m, 4H), 2.67 (s, 1H).

^13^C NMR (151 MHz, CDCl_3_): *δ* = 163.0, 149.7, 144.2, 144.0, 141.5, 141.3, 133.6, 131.9, 129.6, 129.3, 128.3, 126.6, 126.2, 126.0, 125.1.

MS (EI): *m/z* (%) = 416 ([M]^+^, 2), 141 (32), 139 (100), 125 (45), 111 (27), 92 (65).

IR (KBr): ν = 1747, 1594, 1530, 1346, 1210, 1049, 834 cm^–1^.

HRMS (ESI): 417.0305 calcd. for C_19_H_14_O_5_N_2_ClS [M+H]^+^: 417.0307.

##### 2-Nitro-5-(phenylthio)phenyl 4-chlorobenzoate (3)

To a solution of (3-hydroxy-4-nitro)phenylthioether (**4**) (1.4 g, 5.3 mmol) in dry CH_2_Cl_2_ (28 mL) under argon was added triethylamine (1.1 mL, 8.0 mmol). 4-Chlorobenzoyl chloride (0.75 mL, 5.9 mmol) was added and the reaction mixture was stirred at r.t. for 16 h. Water (20 mL) was added and the two phases were separated. The aqueous phase was extracted with CH_2_Cl_2_ (3 x 30 mL) and the combined organic layers were dried over anhydrous magnesium sulfate. After removing the solvents under reduced pressure and purification by column chromatography (Et_2_O/*n*-pentane 20:1) the product was obtained as a light yellow solid (2.0 g, 97%) Mp.: 95 – 96 °C.

^1^H NMR (600 MHz, CDCl_3_): *δ* = 8.09 (d, *J* = 8.6 Hz, 2H), 8.04 (d, *J* = 8.8 Hz, 1H), 7.58 – 7.57 (m, 2H), 7.49 – 7.47 (m, 5H), 7.07 (dd, *J* = 8.8, 2.3 Hz, 1H), 6.98 (d, *J* = 2.0 Hz, 1H).

^13^C NMR (100 MHz, CDCl_3_): *δ* = 163.4, 149.4, 144.7, 140.7, 138.4, 135.0, 131.8, 130.2, 130.0, 129.6, 129.1, 126.4, 123.9, 122.0.

MS (EI): *m/z* (%) = 385 ([M]^+^, 4), 339 (2), 171 (10), 141 (57), 139 (100), 113 (15), 111 (50).

IR (KBr): ν = 3054, 1740, 1580, 1500, 1329, 1063, 741 cm^–1^.

HRMS (ESI): 408.0066, calcd. for C_19_H_12_O_4_NClNaS [M+Na]^+^: 408.0068.

##### 2-Nitro-5-[*N*-(2,2,2-trifluoroacetyl)phenylsulfilimidoyl]phenyl 4-chloro-benzoate (5)

To a mixture of 2-nitro-5-(phenylthio)phenyl 4-chlorobenzoate (**3**) (50 mg, 0.13 mmol), trifluoro­acetamide (29 mg, 0.26 mmol), magnesium oxide (21 mg, 0.52 mmol), Rh_2_(OAc)_4_ (1.4 mg, 2.5 mol%), in CH_2_Cl_2_ (1.3 mL) was added PhI(OAc)_2_ (63 mg, 0.2 mmol).^2^ After stirring at r.t. for 20 h the solvent was removed under reduced pressure, and purification by column chromatography (AcOEt/*n*-pentane 5:1 to 1:1) provided the product as a white solid (53 mg, 82%). Mp.: 48 – 50 °C.

^1^H NMR (400 MHz, CDCl_3_): *δ* = 8.21 (d, *J* = 8.7 Hz, 1H), 8.08 (d, *J* = 8.7 Hz, 2H), 7.89 (d, *J* = 1.9 Hz, 1H), 7.83 – 7.81 (m, 2H), 7.78 (dd, *J* = 6.7, 2.0 Hz, 1H), 7.64 – 7.56 (m, 3H), 7.50 (d, *J* = 8.7 Hz, 2H).

^13^C NMR (100 MHz, CDCl_3_): *δ* = 167.0 (q, *J* = 35.7 Hz), 162.8, 144.9, 144.0, 141.5, 140.7, 133.9, 132.5, 132.0, 130.8, 129.3, 128.3, 127.3, 125.9, 125.5, 124.6, 121.1, 116.8 (q, *J* = 287.7 Hz).

^19^F NMR (376 MHz, CDCl_3_): *δ* = –73.41.

MS (EI): *m/z* (%) = 496 ([M]^+^, 2), 358 (9), 289 (12), 247 (14), 141 (31), 139 (100), 111 (32).

IR (KBr): ν = 1750, 1638, 1593, 1533, 1255, 1205, 1046 cm^–1^.

HRMS (ESI): 518.9984 calcd. for C_21_H_12_O_5_N_2_ClF_3_NaS [M+Na]^+^: 518.9999.

##### 2-Nitro-5-[*N*-(2,2,2-trifluoroacetyl)phenylsulfonimidoyl]phenyl 4-chlorobenz­o­­ate

To a mixture of 2-nitro-5-(phenylsulfinyl)phenyl 4-chlorobenzoate (**7**) (61 mg, 0.15 mmol), trifluoro­acetamide (34 mg, 0.30 mmol), magnesium oxide (25 mg, 0.61 mmol), Rh_2_(OAc)_4_ (1.7 mg, 2.5 mol%) in CH_2_Cl_2_ (1.5 mL) was added PhI(OAc)_2_ (73 mg, 0.23 mmol).^2^ After stirring at r.t. for 16 h the solvent was removed under reduced pressure, and purification by column chromatography (AcOEt/*n*-pentane 5:1 to 1:1) provided the product as a white solid (54 mg, 69%) . Mp.: 52 – 50 °C.

^1^H NMR (400 MHz, CDCl_3_): *δ* = 8.22 (d, *J* = 8.7 Hz, 1H), 8.10 – 8.03 (m, 5H), 7.96 (dd, *J*= 8.7, 2.0 Hz, 1H), 7.76 – 7.72 (m, 1H), 7.67 – 7.63 (m, 1H), 7.53 – 7.50 (m, 2H).

^13^C NMR (151 MHz, CDCl_3_): *δ* = 163.8 (q, *J* = 38.6 Hz), 162.7, 144.9, 144.6, 143.9, 141.6, 135.8, 135.4, 132.0, 130.4, 129.4, 128.0, 127.2, 125.8, 125.5, 125.0.

^19^F NMR (376 MHz, CDCl_3_): *δ* = –75.92.

IR (KBr): ν = 1750, 1689, 1536, 1357, 1155, 1047, 830 cm^–1^.

HRMS (ESI): 534.9940 calcd. for C_21_H_12_O_6_N_2_ClF_3_NaS [M+Na]^+^: 534.9949.

##### 2-Nitro-5-(phenylsulfinyl)phenyl 4-chlorobenzoate (7)

To a mixture of 2-nitro-5-(phenylthio)phenyl 4‑chlorobenzoate (**3**) (200 mg, 0.52 mmol) in acetic acid (2.5 mL) was added aq H_2_O_2_ (30%, 0.24 mL)^3^ and CH_2_Cl_2_ (1 mL). After stirring at r.t. for 18 h, water was added, and the aqueous phase was extracted with CH_2_Cl_2_. The combined organic layers were dried over anhydrous magnesium sulfate and the solvents were removed under reduced pressure. Purification by column chromatography (AcOEt/*n*‑pentane 3:1 to1:1) provided the product as a light yellow solid (157 mg, 75%). Mp.: 106 – 107 °C.

^1^H NMR (600 MHz, CDCl_3_): *δ* = 8.17 (d, *J* = 8.6 Hz, 1H), 8.09 (d, *J* = 8.5 Hz, 2H), 7.75 (d, *J* = 1.7 Hz, 1H), 7.70 – 7.68 (m, 2H), 7.63 (dd, *J* = 6.8, 1.8 Hz, 1H), 7.51 – 7.49 (m, 5H).

^13^C NMR (151 MHz, CDCl_3_): *δ* = 163.1, 153.3, 144.7, 144.0, 142.8, 141.1, 132.3, 131.9, 129.9, 129.2, 126.7, 126.3, 125.0, 122.2, 121.8.

MS (EI): *m/z* (%) = 401 ([M]^+^, 3), 141 (32), 139 (100), 111 (8).

IR (KBr): ν = 3084, 1737, 1586, 1516, 1256, 1051, 834 cm^–1^.

HRMS (ESI): 424.0017 calcd. for C_19_H_12_O_5_NClNaS [M+Na]^+^: 424.0017.

##### (3-Hydroxy-4-nitro)phenylthioether (4)

A mixture of 5-fluoro-2-nitrophenol (300 mg, 1.9 mmol), phenylthiotrimethylsilane (0.44 mL, 2.3 mmol), TBAF (5 mg, 0.02 mmol) and acetonitrile (3.8 mL) was stirred at r.t. for 16 h. The solvent was removed under reduced pressure. After purification by column chromatography (AcOEt/*n*-pentane 100:1 to 50:1) the product was obtained as a yellow solid (413 mg, 82%).

^1^H NMR (400 MHz, CDCl_3_): *δ* = 10.73 (s, 1H), 7.92 (d, *J* = 9.6 Hz, 1H), 7.56 – 7.45 (m, 5 H), 6.67 – 6.64 (m, 2H).

^13^C NMR (100 MHz, CDCl_3_): *δ* = 155.3, 152.8, 135.3, 130.09, 130.1, 129.3, 125.2, 117.9, 115.2.

The corresponding spectroscopic data matched that reported in the literature.^4^

**References**

(1) Miao, J.; Richards, N. G. J.; Ge, H. *Chem. Commun.* **2014**, *50*, 9687.

(2) Okamura, H.; Bolm, C. *Org. Lett.* **2004**, *6*, 1305.

(3) Golchoubian, H.; Hosseinpoor, F. *Molecules* **2007**, *12*, 304.

(4) Liu, C.; Zang, X.; Yu, B.; Yu, X.; Xu, Q. *Synlett* **2011**, 1143.
